# Dermoscopic Features and Their Association With Breslow Thickness of Facial Lentigo Maligna in Koreans: A Multi‐Center Retrospective Study

**DOI:** 10.1111/1346-8138.17822

**Published:** 2025-06-17

**Authors:** Jinie Lee, Ji Su Lee, Jun Young Kim, Jung Min Bae, Jin Park, Je‐Ho Mun

**Affiliations:** ^1^ Seoul National University College of Medicine Seoul Republic of Korea; ^2^ Department of Dermatology Seoul National University College of Medicine Seoul Republic of Korea; ^3^ Institute of Human‐Environment Interface Biology Seoul National University Seoul Republic of Korea; ^4^ Department of Dermatology School of Medicine, Kyungpook National University, Kyungpook National University Hospital Daegu South Korea; ^5^ Department of Dermatology St. Vincent's Hospital, College of Medicine, The Catholic University of Korea Seoul Korea; ^6^ Department of Dermatology Research Institute of Clinical Medicine of Chonbuk National University‐Biomedical Research Institute of Chonbuk National University Hospital Jeonju Korea

**Keywords:** Breslow thickness, dermoscopy, lentigo maligna, lentigo maligna melanoma, pigmented skin lesion

## Abstract

**Background:**

Owing to the rarity of lentigo maligna (LM) and lentigo maligna melanoma (LMM) in East Asians, their dermoscopic features are underreported. The prognosis and management of LM and LMM depend on the Breslow thickness (BT). However, the association between BT and the dermoscopic features of LMM is largely unknown.

**Objectives:**

To report the dermoscopic features of LM/LMM in Koreans and analyze the association between BT and dermoscopic findings of LMM.

**Methods:**

This retrospective study included 46 patients with facial LM/LMM (32 patients had ≤ 1 mm BT and 14 had > 1 mm) collected from three tertiary hospitals in Korea. The frequency of each dermoscopic feature of LM/LMM was assessed according to the BT. Logistic regression analysis was performed to investigate the association between certain dermoscopic patterns and BT in patients with LM/LMM.

**Results:**

Observed dermoscopic patterns in Korean patients with LM/LMM included asymmetrical pigmented follicular openings (100%), asymmetry of the overall shape (97.8%), annular–granular pattern (95.7%), dark rhomboids (95.7%), blotches (78.3%), polychromy (45.7%), blue–white veil (41.3%), thin brown network (36.4%), regression structures (19.6%), and fingerprint pattern (8.7%). Milky‐red areas (32.6%), red rhomboids (26.1%), linear vessels (21.7%), arborizing vessels (8.7%), dotted vessels (2.2%), and hairpin vessels (2.2%) were also observed. Multivariable logistic regression revealed that a blue–white veil (odds ratio [OR], 42.895; 95% confidence interval [CI], 1.878–979.565), red rhomboids (OR, 13.666; 95% CI, 1.070–174.552), and linear vessels (OR, 18.823; 95% CI, 1.357–261.107) were significantly associated with LMM with a BT of > 1 mm. The predictive model (range: 0–7) had a reliable diagnostic value (area under the curve = 0.964).

**Conclusions:**

This study provides an in‐depth analysis of the dermoscopic features of LM/LMM in East Asian patients. Preoperative dermoscopy, which provides BT information, may help determine the appropriate management of LMM.

## 1 Introduction

Lentigo maligna (LM) and lentigo maligna melanoma (LMM) are types of melanoma that arise on chronically photoaged skin and occur predominantly in Caucasian populations. Early detection and prompt surgical management of in situ LM are crucial, as its invasive form (LMM) shares a similar prognosis and malignant potential with other types of invasive melanomas [1]. Although the diagnostic utility of dermoscopy and specific patterns that distinguish LM from benign lesions have been well studied in Western populations, the dermoscopic patterns of LM and LMM among Asians, including Koreans, are largely unknown.

The mortality rate and surgical margins of LMM are affected by tumor thickness. Therefore, the preoperative assessment of the Breslow thickness (BT) is crucial for selecting an appropriate management strategy. Currently, a cut‐off BT of > 1 mm reflects higher tumor staging and wider surgical margins in LMM [2]. Owing to the limited data in the literature, the relationship between invasion depth and dermoscopic findings in LM/LMM warrants further investigation. Therefore, we aimed to report the dermoscopic patterns of facial LM/LMM in Korean patients and analyze the association between BT and these dermoscopic patterns to propose a predictive model for identifying LMM with a BT of > 1 mm.

## 2 Materials and Methods

We collected data of patients with facial LM/LMM who were evaluated using dermoscopy at three tertiary hospitals in Korea: Seoul National University Hospital (SNUH), Jeonbuk National University Hospital (JBNUH), and Kyungpook National University Hospital (KNUH). Histopathological confirmation of the diagnosis and availability of high‐quality clinical and dermoscopic images were the primary inclusion criteria. Scalp and mucosal lesions were excluded because they displayed distinct patterns on dermoscopy. Dermoscopic images were acquired using DermLite II Pro HR, DL3, or DL4 (3Gen, San Juan Capistrano, CA, USA) and an Illuco IDS‐1100 (Illuco Corporation, South Korea) coupled to a digital or mobile phone camera.

### 2.1 Analysis of Demographic and Dermoscopic Features

Clinical and histopathological data, including sex, age at diagnosis, duration, location and diameter of the lesion, lesion number, and BT, were acquired from electronic medical records and histopathological analysis data. Lesions were categorized as LM or LMM. Lentigo maligna melanoma was further subdivided into melanomas with BT of ≤ 1 and > 1 mm.

Based on a review of previous studies, the following dermoscopic criteria were selected and defined: colors (black, dark brown, light brown, gray, red, blue, and white) and polychromy, asymmetry of the total lesion, asymmetrical pigmented follicular openings (semicircles, signet ring‐like circles, gray circles, concentric circles, and target‐like circles), pigmented structures (annular–granular pattern, dark rhomboids, blotches, blue–white veil, regression structures, fingerprint pattern, and thin brown network), vascular structures (red rhomboids, milky‐red areas, dotted, linear, hairpin, and arborizing vessels), and the number of the type of vascular structures (polymorphic vascular pattern). Definitions of the patterns and structures are presented in Table 1 [3, 4, 5, 6, 7, 8]. Each dermoscopic feature was coded as “present” or “absent.” Evaluation was performed by three authors (JL, JSL, and JHM), and any discrepancy was resolved by consensus.

**TABLE 1:** Definitions of dermoscopic patterns and structures.

Melanoma‐specific structure	Definition
**Color**	Colors: black, dark brown, light brown, gray, red, blue, and white were defined as present when agreeably visible in a part of the lesion.
Pattern	
Asymmetry of total lesion	Defined as present when colors or patterns differed between two halves divided by a longitudinal central line, absent only when the lesion appeared completely symmetrical.
Polychromy	Defined as present when five or more of the seven analyzed colors were visible.
Asymmetrical pigmented follicular openings	Pigment associated with adnexal opening that does not uniformly surround the entire opening, or curved lines partially surrounding adnexal openings.
Semicircles	An incomplete pigmented circle that partially surrounds a follicular opening.
Signet ring‐like circles	Pigmented follicular circle with a thicker segment, forming a signet ring shape.
Gray circles	Circular pigmented follicular opening of a gray hue.
Concentric circles	Gray circle within another circle.
Target‐like circles	Dark dot within a circular pigmented follicular opening, excluding hair horizontally sectioned.
Annular–granular pattern	Dots and structureless areas of pigmentation arranged around follicular openings.
Dark rhomboids	Polygonal shapes comprised of brown or gray angulated lines around follicular openings.
Blotches (obliterated follicles)	Dark structureless areas, an extension of dark rhomboids.
Blue–white veil	An irregular blotch of blue hue with an overlying whitish ground‐glass haze.
Regression structures	Areas of multiple fine blue–gray dots (peppering/granularity), or areas whiter than surrounding normal‐appearing skin; these should not be confused with hypo/depigmentation caused by simple melanin loss (Scar‐like depigmentation).
Fingerprint pattern	Light brown thin curved lines that do not interconnect to form a network.
Thin brown network	Grid of interfollicular brown to dark brown, thin lines delimiting relatively uniform‐size circular or oval meshes smaller than follicular openings.
Vascular structure	
Red rhomboids	Lozenge‐shaped vascular pattern occurring in the area separating the hair follicles from the others.
Milky‐red areas	Milky‐white appearance or pinkish structureless areas, consisting of a red vascular blush with no specific vessels.
Dotted vessels	Tiny pinpoint vessels.
Linear vessels	Linear, mildly curved vessels. Considered irregular when different sizes, shapes, and curves with a haphazard distribution; regular when short and fine vessels prevail.
Hairpin vessels	Two parallel linear vessels forming a half‐looped structure.
Arborizing vessels	Large vessels branching into smaller vessels.
Polymorphic vascular patterns	Defined as present when two or more of the six following vascular structures: red rhomboids, milky‐red areas, dotted, linear, hairpin, or arborizing vessels, were visible.

### 2.2 Statistical Analysis

Pearson's chi‐squared or Fisher's exact test was used for univariate analysis to compare dermoscopic features across different invasion depth categories. Crude odds ratios (ORs) with 95% confidence intervals (CIs) were calculated using a univariate logistic regression. For cases where zero counts were present in the contingency tables, the Haldane–Anscombe correction was applied to calculate the ORs and CIs from 2 × 2 contingency tables.

The variables were entered into a multivariable logistic regression model with backward elimination to adjust for possible confounders and to determine independent predictors of LMM with a BT of > 1 mm. Beta coefficients obtained from the multivariable analysis were used to create a predictive model for LMM of BT > 1 mm. Sensitivity and specificity of different cut‐off values were calculated, and a receiver operating characteristic (ROC) curve was generated to evaluate the model's predictive performance. Statistical analyses were performed using IBM SPSS Statistics for Windows version 26.0 (IBM, Armonk, NY, U.S.A). A *p* value of 0 < 0.05 was an indicator of statistical significance in all the analyses.

## 3 Results

### 3.1 Comparison of Demographic and Clinical Characteristics

Forty‐six LM/LMM lesions were analyzed (17 from SNUH, 11 from JBNUH, and 18 from KNUH). They were divided into three groups: LM (22 patients, 47.8%), LMM with BT ≤ 1 mm (10 patients, 21.7%), and LMM with BT > 1 mm (14 patients, 30.4%).

Mean BT for LMM was 2.8 mm (range, 0.2–11.00 mm; standard deviation [SD], 2.9). Mean age of the patients was 65.2 years (range, 35–88 years; SD, 12.5 years), with a mean duration of 6.6 years. Twenty‐eight (60.8%) patients were women, and 34 (73.9%) lesions were located on the cheeks (73.9%). Mean lesion diameter was 22.5 mm (range, 5–60 mm; SD, 11.8 mm).

### 3.2 Dermoscopic Findings of LM/LMM in Korean Populations

The dermoscopic features of LM/LMM are summarized in Table 2. A light brown color was observed in all the patients, followed by dark brown (93.5%), gray (82.6%), black (76.1%), white (42.3%), blue (39.1%), and red (39.1%). Asymmetrical pigmented follicular openings were observed in all the patients. Gray circles were the second most common form (87.0%), followed by semicircles (78.3%), concentric circles (37.0%), target‐like circles (34.8%), and signet ring‐like circles (19.6%). Other observed patterns included overall shape asymmetry (97.8%), annular–granular pattern (95.7%), dark rhomboids (95.7%), blotches (78.3%), polychromy (45.7%), blue–white veils (41.3%), thin brown networks (36.4%), regression structures (19.6%), and fingerprint patterns (8.7%). Among the vascular structures, milky‐red areas were the most common (32.6%), followed by red rhomboids (26.1%), linear vessels (21.7%), and arborizing vessels (8.7%); dotted and hairpin vessels were observed in one (2.2%) patient.

**TABLE 2:** Dermoscopic features of LM and LMM with BT ≤ 1 and > 1 mm.

Feature	Total *n* = 46, *n* (%)	LM *n* = 22, *n* (%)	LMM with BT ≤ 1 mm *n* = 10, *n* (%)	LMM with BT > 1 mm *n* = 14, *n* (%)	*p*
Color					
Black	35 (76.1)	14 (63.6)	9 (90.0)	12 (85.7)	0.231
Dark brown	43 (93.5)	20 (90.9)	10 (100)	13 (92.9)	1.000
Light brown	46 (100)	22 (10)	10 (100)	14 (100)	
Gray	38 (82.6)	19 (86.4)	8 (80.0)	11 (78.6)	0.783
Red	18 (39.1)	4 (18.2)	2 (20.0)	12 (85.7)	**< 0.001**
Blue	18 (39.1)	4 (18.2)	2 (20.0)	12 (85.7)	**< 0.001**
White	19 (41.3)	4 (18.2)	2 (20.0)	13 (92.9)	**< 0.001**
Pattern					
Asymmetry of total lesion	45 (97.8)	21 (95.5)	10 (100)	14 (100)	1.000
Polychromy	21 (45.7)	6 (27.3)	2 (20.0)	13 (92.9)	**< 0.001**
Asymmetrical pigmented follicular openings	46 (100)	22 (10)	10 (100)	14 (100)	
Semicircles	36 (78.3)	17 (77.3)	8 (80.0)	11 (78.6)	1.000
Signet ring‐like circles	9 (19.6)	5 (22.7)	3 (30.0)	1 (7.1)	0.339
Gray circles	40 (87.0)	21 (95.5)	8 (80.0)	11 (78.6)	0.235
Concentric circles	17 (37.0)	8 (36.4)	4 (40.0)	5 (35.7)	1.000
Target‐like circles	16 (34.8)	8 (36.4)	4 (40.0)	4 (28.6)	0.850
Annular–granular pattern	44 (95.7)	22 (100)	10 (100)	12 (85.7)	0.131
Dark rhomboids	44 (95.7)	21 (95.5)	10 (100)	13 (92.9)	1.000
Blotches	36 (78.3)	16 (72.7)	8 (80.0)	12 (85.7)	0.735
Blue–white veil	19 (41.3)	4 (18.2)	2 (20.0)	13 (92.9)	**< 0.001**
Regression structures	9 (19.6)	2 (9.1)	1 (10.0)	6 (42.9)	0.052
Fingerprint pattern	4 (8.7)	3 (13.6)	1 (10.0)	0 (0.0)	0.418
Thin brown network	16 (34.8)	7 (31.8)	4 (40.0)	5 (35.7)	0.923
Vascular structure					
Red rhomboids	12 (26.1)	2 (9.1)	1 (10.0)	9 (64.3)	**0.** **001**
Milky‐red areas	15 (32.6)	3 (13.6)	2 (20.0)	10 (71.4)	**0.** **001**
Dotted vessels	1 (2.2)	0 (0.0)	0 (0.0)	1 (7.1)	0.522
Linear vessels	10 (21.7)	0 (0.0)	1 (10.0)	9 (64.3)	**< 0.001**
Hairpin vessels	1 (2.2)	0 (0.0)	0 (0.0)	1 (7.1)	0.522
Arborizing vessels	4 (8.7)	0 (0.0)	0 (0.0)	4 (28.6)	**0.** **007**
Polymorphic vascular patterns	12 (26.1)	1 (4.5)	1 (4.5)	10 (71.4)	**< 0.001**

*Note:* Bold font indicates statistical significance (*p* < 0.05).

Abbreviations: BT, Breslow thickness; LM, lentigo maligna; LMM, lentigo maligna melanoma.

**FIGURE 1:**
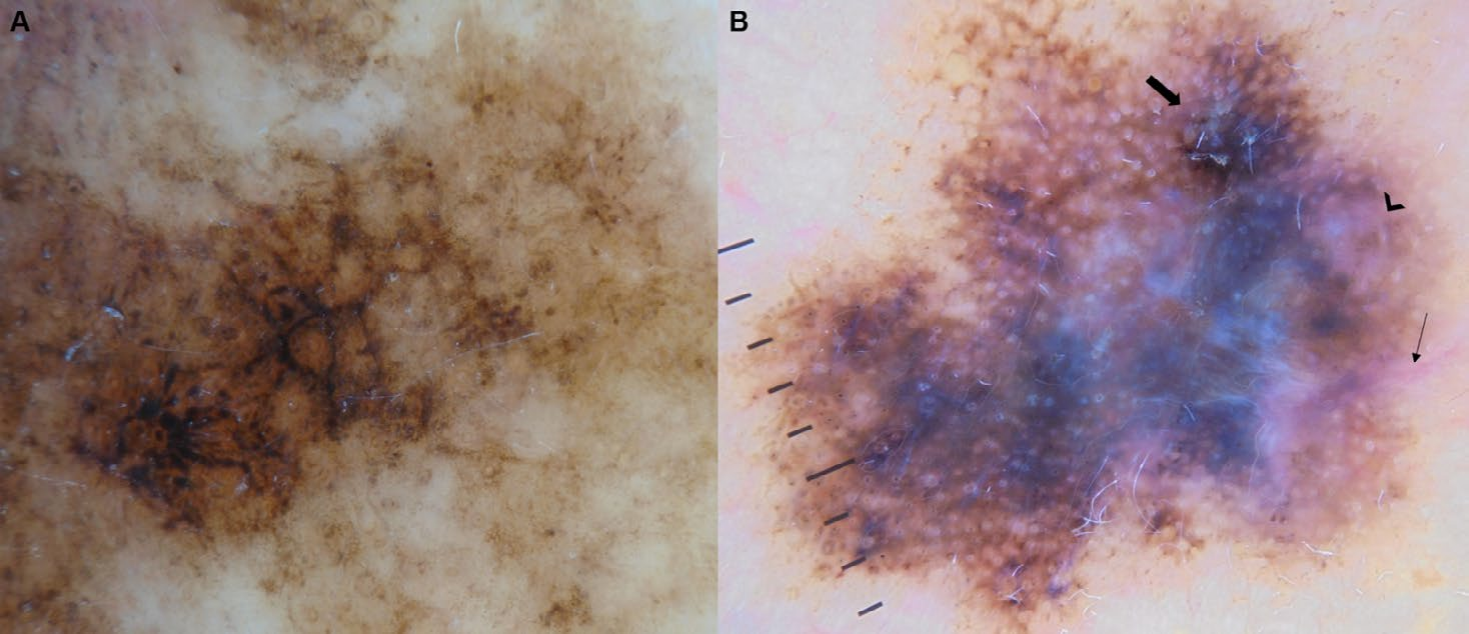
Dermoscopic findings of lentigo maligna and lentigo maligna melanoma with Breslow thickness > 1 mm. (A) Lentigo maligna showing an annular–granular pattern, gray circles, and dark rhomboids. (B) Lentigo maligna melanoma with a Breslow thickness of 3.5 mm showing milky‐red areas (arrowhead), central blue–white veil, linear vessels (narrow arrow), and blotches (thick arrow).

Table 2 shows the frequencies of dermoscopic features across the three groups: LM, LMM with BT ≤ 1 mm, and LMM with BT > 1 mm. When comparing the three groups, lesions with greater depth showed a significantly higher prevalence of red (*p* < 0.001), blue (*p* < 0.001), and white (*p* < 0.001). Polychromy (*p* < 0.001), blue–white veils (*p* < 0.001), and polychromy were significantly associated with a greater invasion depth. The vascular structures of the red rhomboids (*p* = 0.001), milky‐red areas (*p* = 0.001), linear vessels (*p* < 0.001), arborizing vessels (*p* = 0.007), and polymorphic vascular patterns (*p* < 0.001) were also more frequently observed in the deeper group (Figure 1).

### 3.3 Logistic Regression Analysis of Dermoscopic Findings Associated With an LMM of > 1 mm

Table 3 shows the results of the univariate logistic regression analysis. LMM with BT > 1 mm was significantly associated with the colors red (*p* < 0.001; OR, 26.00), blue (*p* < 0.001; OR, 26.00), and white (*p* < 0.001; OR 56.33). Blue–white veil (*p* < 0.001; OR 56.33), polychromy (*p* = 0.001; OR, 39.00), regression structures (*p* = 0.015; OR, 7.25), red rhomboids (*p* = 0.001; OR, 17.40), polymorphic vascular patterns (*p* < 0.001; OR, 37.50), milky‐red areas (*p* = 0.001; OR, 13.50), and linear vessels (*p* = 0.001; OR, 55.80) were indicative of LMM with a BT of > 1 mm.

**TABLE 3:** Univariable analysis of dermoscopic features associated with LMM with BT > 1 mm.

Feature	LM/LMM of BT ≤ 1 mm *n* = 32, *n* (%)	LMM of BT > 1 mm *n* = 14, *n* (%)	Univariable analysis	
			Crude OR (95% CI)	*p*
Color				
Black	23 (71.9)	12 (85.7)	2.348 (0.436–12.644)	0.320
Dark brown	30 (93.8)	13 (92.9)	0.867 (0.072–10.423)	0.910
Light brown	32 (100)	14 (100)	—	—
Gray	27 (84.4)	11 (78.6)	0.679 (0.138–3.342)	0.634
Red	6 (18.8)	12 (85.7)	26.000 (4.562–148.184)	**< 0.001**
Blue	6 (18.8)	12 (85.7)	26.000 (4.562–148.184)	**< 0.001**
White	6 (18.8)	13 (93.9)	56.333 (6.123–518.276)	**< 0.001**
Pattern				
Asymmetry of total lesion	31 (96.9)	14 (100)	1.381 (0.053–35.993)	**1.000**
Polychromy	8 (25.0)	13 (92.9)	39.000 (4.384–346.971)	**0.001**
Asymmetrical pigmented follicular openings	32 (100)	14 (100)	—	—
Semicircles	25 (78.1)	11 (78.6)	1.027 (0.223–4.728)	0.973
Signet ring‐like circles	8 (25.0)	1 (7.1)	0.231 (0.026–2.053)	0.189
Gray circles	29 (90.6)	11 (78.6)	0.379 (0.066–2.170)	0.276
Concentric circles	12 (37.5)	5 (35.7)	0.926 (0.251–3.420)	0.908
Target‐like circles	12 (37.5)	4 (28.6)	0.667 (0.171–2.604)	0.560
Annular–granular pattern	32 (100)	12 (85.7)	0.077 (0.003–1.717)	0.088
Dark rhomboids	31 (96.9)	13 (92.9)	0.419 (0.024–7.224)	0.550
Blotches	24 (75.0)	12 (92.9)	2.000 (0.366–10.919)	0.423
Blue–white veil	6 (18.8)	13 (92.9)	56.333 (6.123–518.276)	**< 0.001**
Regression structures	3 (9.4)	6 (42.9)	7.250 (1.476–35.611)	**0.015**
Fingerprint pattern	4 (12.5)	0 (0.0)	0.218 (0.011–4.340)	0.298
Thin brown network	11 (34.4)	5 (35.7)	1.061 (0.285–3948)	0.930
Vascular structure				
Red rhomboids	3 (9.4)	9 (64.3)	17.400 (3.461–87.481)	**0.001**
Milky‐red areas	5 (15.6)	10 (71.4)	13.500 (3.007–60.605)	**0.001**
Dotted vessels	0 (0.0)	1 (7.1)	7.222 (0.276–188.697)	0.304
Linear vessels	1 (3.1)	9 (64.3)	55.800 (5.755–541.015)	**0.001**
Hairpin vessels	0 (0.0)	1 (7.1)	7.222 (0.276–188.697)	0.304
Arborizing vessels	0 (0.0)	4 (28.6)	27.857 (1.382–561.482)	**0.006**
Polymorphic vascular patterns	2 (6.3)	10 (71.4)	37.500 (5.943–236.614)	**< 0.001**

*Note:* Bold font indicates statistical significance (*p* < 0.05).

Abbreviations: BT, Breslow thickness; CI, confidence interval; LM, lentigo maligna; LMM, lentigo maligna melanoma; OR, Odds ratios.

**FIGURE 2:**
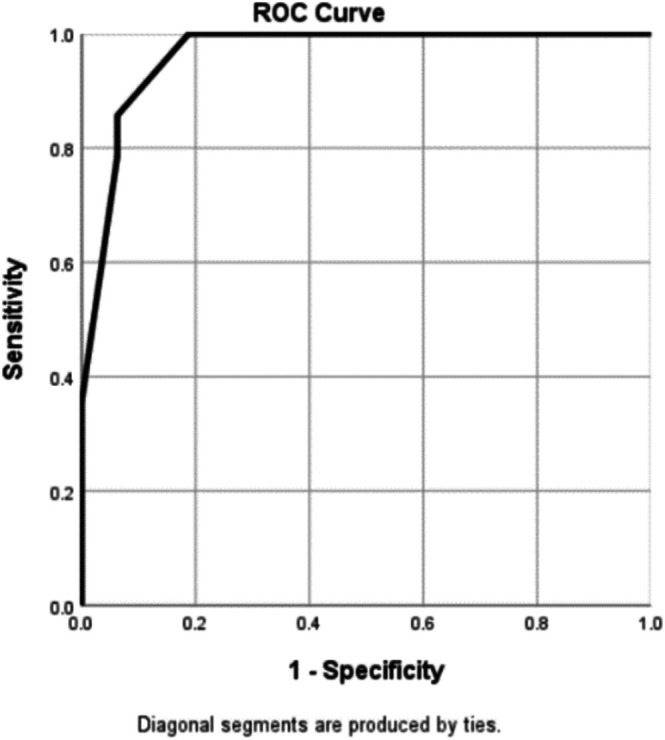
Receiver operating characteristic curve for a predictive model for differentiating facial lentigo maligna melanoma with Breslow thickness of ≤ 1 and > 1 mm.

Multivariate logistic regression was conducted using dermoscopic patterns that demonstrated significance in the univariate logistic regression analysis. Specific features of blue–white veils (OR, 42.90; 95% CI, 1.88–979.57), red rhomboids (OR, 13.67; 95% CI: 1.07–174.55), and linear vessels (OR, 18.82; 95% CI, 1.36–261.11) were predictive factors for LMM with BT of > 1 mm (Table 4). The coefficients of these three features were used to create a predictive model for distinguishing between facial LMM with a BT of > 1 mm and thinner lesions. The model scores were between 0 and 7, with points assigned as follows: three for the blue–white veil and two points each for the red rhomboids and linear vessels. The sensitivity and specificity of each cut‐off score are listed in Table S1. A cut‐off score of 3 demonstrated a sensitivity of 100% and specificity of 81.2%, whereas a cut‐off score of 4 demonstrated a sensitivity of 85.7% and specificity of 93.7%, with an area under the curve (AUC) of the ROC curve being 0.964 (Figure 2).

**TABLE 4:** Multivariable analysis and risk score in the predictive model for LMM of BT > 1 mm.

Variables	Multivariable logistic regression		*β* Coefficient	Risk score^a^ for > 1 mm
	OR (95% CI)	*p*		
Blue–white veil	42.895 (1.878–979.565)	0.019	3.759	3
Red rhomboids	13.666 (1.070–174.552)	0.044	2.615	2
Linear vessels	18.823 (1.357–261.107)	0.029	2.935	2

Abbreviations: BT, Breslow thickness; CI, confidence interval; LMM, lentigo maligna melanoma; OR, odds ratio.

^a^ Scores were given by rounding the ratio of the *β* coefficient to the nearest integer‐ 1.

## 4 Discussion

The incidence of LM and LMM is lower in East Asians than that in Caucasians, and studies on the dermoscopic characteristics of these melanomas in East Asians are lacking [9]. Distinct skin characteristics and pigmentation in East Asians present unique clinical and dermoscopic features compared with those in Caucasians, requiring specialized methods for melanoma detection and treatment [10].

Our study provides an in‐depth cohort analysis of the dermoscopic features of LM/LMM in Korean patients with the largest number of cases reported to date. We observed differences in the clinical presentation of LM/LMM between Western and Asian patients. A study on LM/LMM based in the United States reported a mean overall clinical diameter of 11.4 mm (SD, 8.3 mm; range, 2–56 mm) [11], while our data showed a mean diameter of 22.52 mm (SD, 11.82 mm; range, 5–60 mm). Our previous study suggested that the diagnosis of LM/LMM in South Korea is underreported [12]. These data underscore the importance of understanding the ethnic variations in melanoma characteristics to enhance early detection and accurate diagnosis.

We found an association between BT and dermoscopic patterns in LM/LMM, indicating the essential role of dermoscopy in non‐invasive assessment. The BT is a critical factor in melanoma staging and directly influences treatment decisions and prognosis. A cut‐off value of 1 mm delineates tumors with significantly different clinical outcomes. Melanomas with a BT of ≤ 1 mm have a five‐year survival rate exceeding 90%. However, melanomas with a BT of > 1 mm fall into at least the T2 category, where the five‐year survival rates vary between 50% to 90%, depending on tumor thickness, ulceration, and mitotic rate [13]. This distinction can also be extended to surgical management practices. Elective lymph node dissections are generally not recommended for thin melanomas with a BT of ≤ 1 mm, as the likelihood of regional nodal involvement is low [14]. More extensive surgical intervention is required for thicker tumors, and the local surgical design is also defined according to tumor thickness. Additionally, as the face is a cosmetically sensitive area, performing a wide excision of ≥ 1 cm can often result in surgically challenging cases.

Because skin biopsy is invasive and may only partially capture the lesion's characteristics, dermoscopic evaluation of the entire lesion can provide a more comprehensive overview of melanoma thickness and assist in non‐invasive treatment planning. Preoperative prediction of BT in melanoma using dermoscopy has been reported in several studies (Table 5) [15, 16, 17, 18, 19, 20, 21, 22, 23]. However, previous studies included very few patients with LMM (none or two 1.6% patients) or excluded facial lesions altogether [15, 16, 19]. The results of these studies can be summarized as showing patterns related to atypical vascularity, which were indicative of deeper melanomas in eight of nine studies involved [15, 16, 17, 18, 19, 20, 21, 23]. Additionally, structures such as milky‐red areas and corkscrew vessels were also associated with thicker melanomas [16, 19, 20]. Regarding pigmentation, blue–gray areas or blue–white veils were found to be significant in seven of nine studies [15, 16, 17, 18, 19, 21, 22].

**TABLE 5:** Summary of studies on the association of Breslow thickness with dermoscopic patterns of melanoma.

Reference/Year	Number of cases	Melanoma subtype (%)	Location (%)	Cut‐off (mm)	Depth (*n*)	Results of significance
Argenziano et al. (1997)	72	Not specified	Not specified	< 0.76	thin^a^ (41) thick^a^ (31)	Blue–gray area and the presence of a vascular pattern (*p* < 0.001) were associated with thick melanoma, while a pigment network (*p* = 0.002) was associated with thin melanoma.
Argenziano et al. (1999)	122	Not specified	Not specified	< 0.76 > 1.50	thin (72) intermediate (31) thick (19)	Combination of palpability, a diameter of 15 mm or more, blue–gray area, and atypical vascular pattern (linear, dotted, globular structure) helped prediction of thick melanomas.
Stante et al. (2001)	84	SSM (96.4), NM (2.4), AM (1.2), LM(M) (0.0)	Not specified	< 0.76 > 1.50	in situ (17) thin (32) intermediate (26) thick (9)	There was correlation between thick melanomas and radial streaming (*p* = 0.017), atypical vascular pattern (*p* = 0.030), and gray–blue areas (*p* < 0.001). Thinner melanomas were associated with irregular pigment network (*P* = 0.001).
Mun et al. (2018)	75	AM	Acral	≤ 2.0	in situ (25) thin (17) thick (33)	Red (*P* < 0.001; OR, 16.482), blue (*p* = 0.011; OR, 7.092), white (*p* = 0.031; OR, 5.048) color and blue–white veils (*p* = 0.010; OR, 9.605), atypical vascular patterns (*p* < 0.001; OR, 34.589), and ulceration (*p* = 0.034; OR, 5.084) was indicative of thick acral melanoma.
Sgouros et al. (2020)	254	NM	Trunk, Limb, h/n, acral, mucosal	≤ 2.0	thin (69) thick (96) non‐melanoma (89)	Serpentine vessels (*p* = 0.008; OR, 0.3) were more common in thick nodular melanomas, while thin melanomas showed irregular brown dots and globules (*p* = 0.037; OR, 1.8).
Podolec et al. (2020)	81	Not specified	Trunk (45.68), Limb (37.03) Head (17.28)	≤ 1.0	in situ (22) thin (44) thick (15)	Pseudopods (*p* = 0.047; OR, 0.23) and multicomponent pattern (*p* = 0.007; OR, 0.25) were more common in invasive melanomas. White regression structures (*p* = 0.041) were specific for thick melanomas.
Rodríguez‐Lomba et al. (2021)	245	Not specified	Exclusion of facial, acral, genital, mucosal lesions.	< 0.8	in situ (52) thin (98) thick (95)	Red–pink (OR, 4.641; 95% CI, 2.622–8.242), blue–gray (3.743; 2.044–6.853) and white (1.123; 0.632–1.965) color, blue–white veil (8.446; 4.568–15.67), shiny white streaks (2.913; 1.702–4.973), blue–black pigmentation (4.149; 1.724–9.983), milky‐red areas(4.668; 2.395–9.096), irregular vessels(3.796; 2.102–6.855), pseudolacunae (15.653; 4.549–53.854), ulceration (16.917; 6.326–45.265) and rainbow pattern (7.296; 3.018–17.687) were associated with thick melanomas. Thin melanomas were associated with atypical pigment network (0.505; 0.295–0.856), regression (0.408; 0.238–0.717), and hypopigmented areas (0.372; 0.149–0.950).
Martínez‐Piva et al. (2021)	215	SSM (74), NM (22), AM (2.4), LM(M) (1.6)	Trunk (52.1) Lower limb (25.6) Upper limb (12.1) Head/neck (10.2)	< 1.0	in situ (88) thin (73) thick (54)	Whitish blue veil (*p* < 0.001), white shiny structures (*p* < 0.001), and milky‐red areas (*p* = 0.003) were significantly indicative of thick melanomas.
Avila et al. (2024)	43	SSM (88.6), LM(M) (11.6)	Trunk (46.5), Lower limb (23.3) Upper limb (18.6) Head/neck (11.6)	< 1.0	in situ (21) thin (16) thick (6)	Radial streaming (*p* = 0.009) and blue–white veil (*p* = 0.046) were more frequent in thick melanomas than in thin melanomas. Invasive melanomas more commonly had atypical vessels (*p* = 0.018), white scar areas (*p* = 0.021), blue–white veil (*p* = 0.002), and shiny white structures (*p* < 0.001)

Abbreviations: AM, acral melanoma; CI, confidence interval; LM(M), lentigo maligna (melanoma); NM, nodular melanoma; OR, Odds ratios; SSM, superficial spreading melanoma.

^a^ Thin melanomas denote lesions with an invasion depth (equal to or) smaller than the cut‐off (mm) used in each study, while lesions (equal to or) thicker than the cut‐off number were classified as thick melanomas.

**FIGURE 3:**
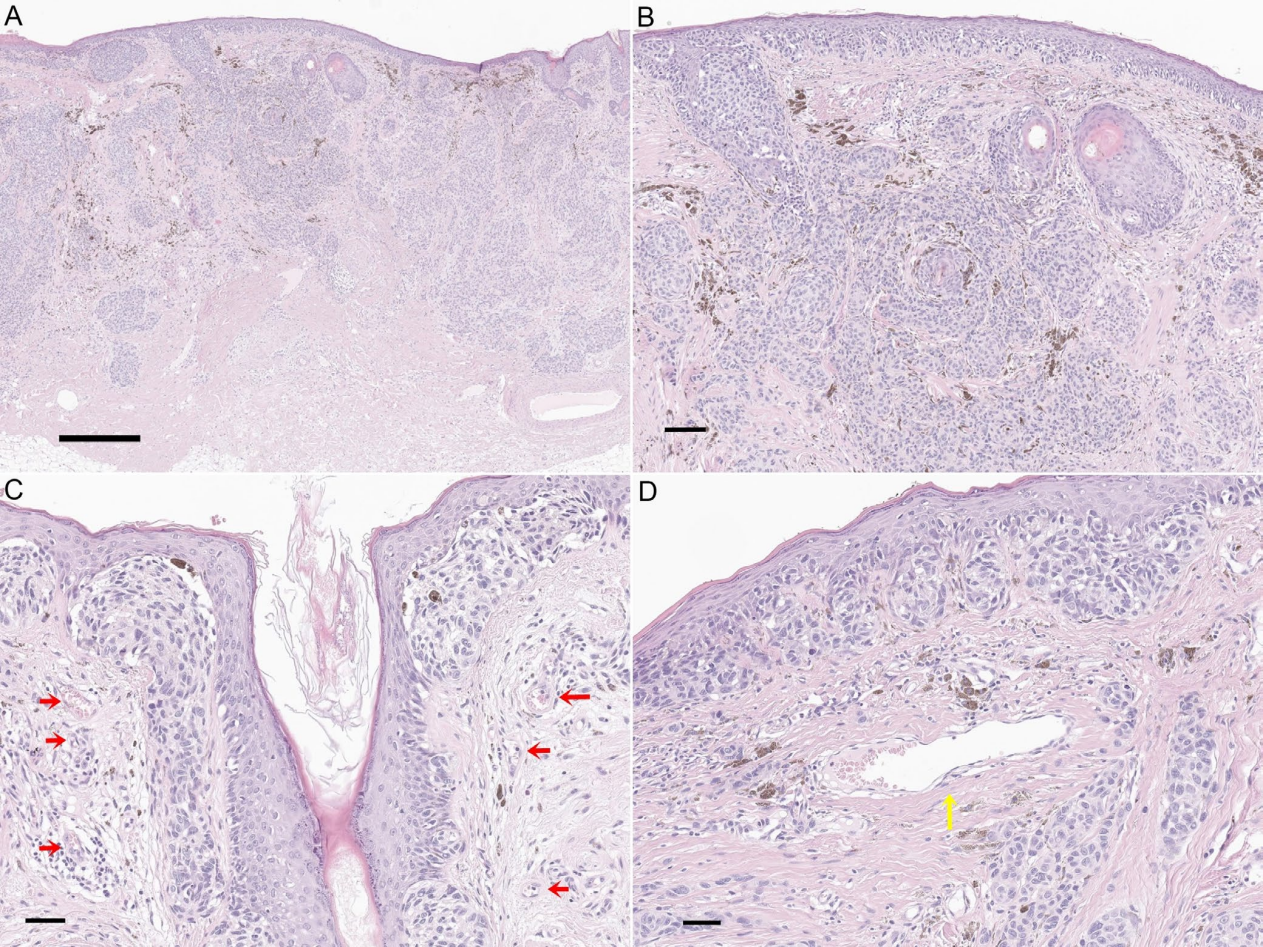
Histopathologic correlates of key dermoscopic features in lentigo maligna melanoma, including blue–white veil, red rhomboids, and linear vessels. (A, B) Lentigo maligna melanoma showing abundant pigmented atypical melanocytes and melanophages in the superficial dermis, corresponding to the dermoscopic feature of a blue–white veil. (A) Low‐power view (scale bar = 500 μm); (B) higher magnification (scale bar = 100 μm). (C) Increased perifollicular capillaries around hair follicles, reflecting the dermoscopic feature of red rhomboids. (Scale bar = 50 μm). (D) Horizontally oriented ectatic capillary in the superficial dermis, corresponding to the dermoscopic feature of linear vessels. (Scale bar = 50 μm).

We found that specific dermoscopic features, including blue–white veils (OR 42.895), red rhomboids (OR. 13.666), and linear vessels (OR 18.823), were significantly associated with LMM with BT > 1 mm. Histologically, the blue–white veil corresponds to densely pigmented melanophages or melanoma cell nests located in the dermis, with or without compact orthokeratosis (Figure 3). The resulting opaque blue hue is attributed to short‐wavelength light backscatter, known as the Tyndall effect [23]. Red rhomboids reflect tumor‐induced vascular meshes surrounding follicular units, with histologic sections showing increased capillary proliferation around hair follicles. Linear irregular vessels are indicative of superficial dermal neoangiogenesis [24]. Histologically, these correspond to slender, horizontally oriented capillaries within the dermis. In invasive melanoma, linear vessels of irregular size and distribution have also been demonstrated using dynamic optical coherence tomography (OCT) [25]. These findings align with the understanding that as vertical tumor growth occurs, there is a corresponding increase in pigmentation and vascular changes. The predictive model developed from these features achieved high diagnostic accuracy with an AUC of 0.964, providing a practical tool for assessing the likelihood of BT > 1 mm in the LMM.

The limitations of this study are its retrospective design and exclusion of extrafacial lesions. Additionally, our study primarily included older patients. A recent study showed that LM/LMM in younger populations often exhibit fewer dermoscopic features, such as perifollicular pigmentation, which hampers early diagnosis [26]. This suggests that diagnostic strategies should consider factors, such as age and ethnicity, which can influence the dermoscopic features. It would be an intriguing topic to investigate whether there are dermoscopic differences in LM and LMM between Asians and Caucasians after adjusting for tumor thickness. Therefore, larger prospective studies that include stratified age groups and various ethnic backgrounds are required to validate and expand upon our findings.

In conclusion, this study demonstrated the dermoscopic patterns of LM/LMM in East Asian patients. Our study, in conjunction with previous findings on LM and LMM in other ethnic groups, highlights both the similarities and variations in their presentations across different demographics. Dermoscopic features, including blue–white veils, red rhomboids, and linear vessels, were predictive indicators of thicker LMM (BT > 1 mm). Preoperative dermoscopic examination can provide a non‐invasive and comprehensive assessment of the strategic management of facial LMM.

## Ethics Statement

The authors declare that they obtained a written informed consent from the patients included in the article and that this report does not contain any personal information that could lead to their identification. The research was reviewed and approved by the Institutional Review Board of Seoul National University Hospital (H‐2301‐071‐1394).

## Conflicts of Interest

The authors declare no conflicts of interest.

## Supporting information


**Table S1.** Sensitivity and specificity of each cut‐off score in the predictive model for LMM with BT > 1 mm.

## Data Availability

The data that support the findings of this study are available from the corresponding author upon reasonable request.
