# Supplementary material for: Dermoscopic Features and Their Association With Breslow Thickness of Facial Lentigo Maligna in Koreans: A Multi‐Center Retrospective Study
Source: J Dermatol. 2025 Jun 17;52(8):1243–54. doi: 10.1111/1346-8138.17822 (PMC12315610; doi:10.1111/1346-8138.17822)
Supplement: Supplementary file 1 — Table S1. Sensitivity and specificity of each cut‐off score in the predictive model for LMM with BT > 1 mm. [file JDE-52-1243-s001.docx]

**Supplemental Material**

| **Supplementary Table 1.** Sensitivity and specificity of each cut-off score in the predictive model for LMM with BT >1 mm | | | |
| --- | --- | --- | --- |
| **Cut-off Score** | **Sensitivity** | **Specificity** | **False positive** |
| 0 | 100.0% | 0.0% | 100.0% |
| 2 | 100.0% | 75.0% | 25.0% |
| 3 | 100.0% | 81.2% | 18.8% |
| 4 | 85.7% | 93.7% | 6.3% |
| 5 | 78.6% | 93.7% | 6.3% |
| 7 | 35.7% | 100.0% | 0.0% |
| Abbreviations: LMM, Lentigo maligna melanoma; BT, Breslow thickness | | | |
